# Uptake, biotransformation and elimination of selected pharmaceuticals in a freshwater invertebrate measured using liquid chromatography tandem mass spectrometry

**DOI:** 10.1016/j.chemosphere.2017.05.083

**Published:** 2017-09

**Authors:** Thomas H. Miller, Nicolas R. Bury, Stewart F. Owen, Leon P. Barron

**Affiliations:** a Analytical & Environmental Sciences Division, Faculty of Life Sciences and Medicine, King's College London, 150 Stamford Street, London, SE1 9NH, United Kingdom; b Division of Diabetes and Nutritional Sciences, Faculty of Life Sciences and Medicine, King's College London, Franklin Wilkins Building, 150 Stamford Street, London, SE1 9NH, United Kingdom; c AstraZeneca, Global Environment, Alderley Park, Macclesfield, Cheshire, SK10 4TF, United Kingdom

**Keywords:** Biotransformation, Pharmaceuticals, *Gammarus pulex*, Bioconcentration

## Abstract

Methods were developed to assess uptake and elimination kinetics in *Gammarus pulex* of nine pharmaceuticals (sulfamethazine, carbamazepine, diazepam, temazepam, trimethoprim, warfarin, metoprolol, nifedipine and propranolol) using targeted LC-MS/MS to determine bioconcentration factors (BCFs) using a 96 h toxicokinetic exposure and depuration period. The derived BCFs for these pharmaceuticals did not trigger any regulatory thresholds and ranged from 0 to 73 L kg^−1^ (sulfamethazine showed no bioconcentration). Metabolism of chemicals can affect accurate BCF determination through parameterisation of the kinetic models. The added selectivity of LC-MS/MS allowed us to develop confirmatory methods to monitor the biotransformation of propranolol, carbamazepine and diazepam in *G. pulex*. Varying concentrations of the biotransformed products; 4-hydroxypropranolol sulphate, carbamazepine-10,11-epoxide, nordiazepam, oxazepam and temazepam were measured following exposure of the precursor compounds. For diazepam, the biotransformation product nordiazepam was present at higher concentrations than the parent compound at 94 ng g^−1^ dw. Overall, the results indicate that pharmaceutical accumulation is low in these freshwater amphipods, which can potentially be explained by the rapid biotransformation and excretion.

## Highlights

• Bioconcentration factors of 9 pharmaceuticals in *G. pulex* ranged from 0 to 73 L kg^−1^
• *G. pulex* are capable of pharmaceutical biotransformation.
• Five pharmaceutical metabolites were determined up to 94.5 ng g^−1^
• Biotransformation can affect BCF_total_ estimates if metabolites are not accounted for.

## Graphical abstract

**Figure:**
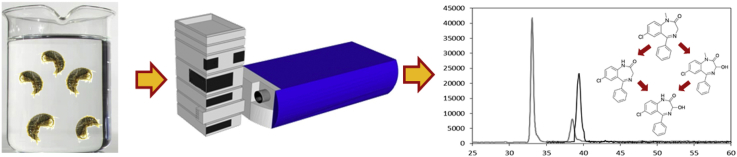

## 1 Introduction

Extensive research into organic environmental micropollutants has enabled the elucidation of the mechanisms for the uptake and accumulation in biota (Barber et al., 1988, Barber et al., 1991, Mackay and Fraser, 2000). Uptake was mainly considered to occur by passive diffusion across cellular membranes and traditional models relied heavily on physico-chemical properties such as octanol-water partition coefficients (log*P*) to describe and predict xenobiotic concentrations in biota (Kanazawa, 1981, Neely et al., 1974, Veith et al., 1979). Such earlier works often focussed on neutral compounds (Fu et al., 2009, Klosterhaus et al., 2013, Wu et al., 2013), but more recently identified micropollutant classes, such as pharmaceuticals, are somewhat different in that they are often ionisable and have a wider range of molecular polarity. Additional mechanisms such as ion trapping, carrier mediated transport and partitioning to non-lipid components (protein binding) could also influence the accumulation of pharmaceutical residues in the environment (Fu et al., 2009, Klosterhaus et al., 2013, Stott et al., 2015). As most of the reported work has focussed on vertebrates such as fish (Gobas et al., 1986, Kanazawa, 1981, Spacie and Hamelink, 1982, Veith et al., 1979), the bioaccumulation of compounds in invertebrates is not well understood.

The OECD 305 guidelines are widely used for estimating the bioconcentration factor (BCF) or bioaccumulation factor (BAF) in fish and have also been applied to invertebrates, such as bivalves and amphipods (Ashauer et al., 2006, Ashauer et al., 2010, Meredith-Williams et al., 2012, OECD,, Sordet et al., 2016). Estimations using these guidelines can be determined using steady-state or kinetic measurements. Kinetic measurement estimates are based on non-linear regression to generate uptake (*k*_1_) and elimination rate constants (*k*_2_) and used together to estimate BCF/BAF. Models assume that rate constants do not change. However, recent investigations in our laboratory showed that the OECD model led to significant lack-of-fits for measured data in invertebrates (Miller et al., 2016). The lack-of-fits were shown to arise from a potentially decreasing *k*_1_ trend over time for a proportion of compounds tested. This finding was significant as such models could lead to under/over estimation of BCF/BAFs during risk assessment of chemicals in invertebrates. Possible causes for the decreases in *k*_1_ could be related to several factors such as growth, metabolism and sorption processes. Metabolism in particular, is generally not considered in bioconcentration studies. Many analytical methods rely on measurement of total radioactivity of a labelled compound using liquid scintillation counting (LSC) (Arnot and Gobas, 2006) as an alternative to confirmatory analytical tools based on liquid chromatography (LC) or gas chromatography (GC) coupled to mass spectrometry (MS). However for small freshwater invertebrates, only a few published LC or GC-MS-based methods exist for parent compound determination (Grabicova et al., 2015, Inostroza et al., 2016, Miller et al., 2015, Sordet et al., 2016). A reason for the small number of published methods is that sensitivity at environmentally relevant concentrations is often challenging due to their small size. Similarly, simply increasing the sample mass to be extracted (via pooling of individuals) is often undesirable due to extra MS signal suppression or enhancement effects caused by the matrix. Therefore, a delicate balance is required to ensure sufficient MS sensitivity and that reliable quantifications can be performed. In the absence of confirmatory analytical methods, biotransformation during the exposure period could have a significant effect on invertebrate BCF/BAF estimation using scintillation counting methods (de Wolf et al., 1992, Oliver and Niimi, 1985, Opperhuizen and Sijm, 1990). Xenobiotics can also induce or inhibit their own metabolism or the metabolism of other compounds that will affect the clearance rate and hence the BCF/BAF (Golan et al., 2011). Moreover, as standardised methods to measure metabolic products and the kinetics of biotransformation currently do not exist, it is difficult to assess the influence of biotransformation in accumulation (Cowan-Ellsberry et al., 2008). Several authors have applied *in vitro* intrinsic clearance rates to extrapolate to whole body biotransformation rates for predictive BCF modelling (Arnot et al., 2008, Nichols et al., 2013). However, these extrapolations measure only the loss of parent compound to predict whole body biotransformation. Thus, *in vitro* clearance rates may not reliably reflect whole body metabolic rates (Nichols et al., 2006).

To date only three studies have measured biotransformation products and their associated toxicokinetics in invertebrates (Ashauer et al., 2012, Jeon et al., 2013b, Rösch et al., 2016). Two of these works used LC coupled to high resolution MS (HRMS) or LC with a radioactivity detector to model the uptake and elimination profiles of organic micropollutants and their biotransformation products in *G. pulex* (Ashauer et al., 2012, Rösch et al., 2016). Ashauer et al. showed that the measurement of biotransformation improved the accuracy of BCF estimates when compared to estimates using total radioactivity counts (Ashauer et al., 2012). A further constraint to the study of xenometabolism is that *a priori* knowledge of biotransformation products in aquatic organisms is lacking leading to difficulty when developing targeted analytical methods (Celiz et al., 2009). Current methods have focussed on the determination of organic pollutants in fish, with little attention given to invertebrates or pharmaceutical biotransformation. Therefore, it is essential that methods are developed that can determine pharmaceutical biotransformation products to more reliably assess the affect metabolism has on bioconcentration models.

The aim of this work was to assess the bioconcentration of a selection of nine pharmaceuticals in *G. pulex* using targeted LC-MS/MS methods described in (Miller et al., 2015). In this regard, the method developed for pharmaceutical occurrence was used for the determination of selected known pharmaceutical biotransformation products of carbamazepine, diazepam and propranolol. The method showed good performance in terms of linearity, recovery, precision and robustness. In particular, BCFs were estimated using the OECD 305 guidelines and kinetic parameters were checked for constancy over time. Finally, the optimised LC-MS/MS method was used for the identification and determination of biotransformation products of propranolol, carbamazepine and diazepam. As few published works have studied pharmaceutical bioconcentration and biotransformation, the work presented herein addresses the knowledge gaps concerning their bioaccumulation and biotransformation in invertebrates at environmentally relevant concentrations using a minimised test design.

## 2 Materials and methods

### 2.1 Reagents, chemicals and consumables

HPLC grade methanol, acetonitrile, acetone, ethyl acetate, dichloromethane and dimethyldichlorosiloxane were purchased from Fischer Scientific (Loughborough, UK). Analytical grade ammonium acetate was sourced from Sigma-Aldrich (Dorset, UK). Propranolol hydrochloride, warfarin, sulfamethazine, carbamazepine, nimesulide, (±)-metoprolol (+)-tartrate salt, temazepam, diazepam, nifedipine, oxazepam, nordiazepam, carbamazepine-10,11-epoxide, and sulfamethazine were all obtained from Sigma-Aldrich (Steinheim, Germany). Trimethoprim, was ordered from Fluka (Buchs, Switzerland). Stable isotope-labelled standards including carbamazepine-*d*_10_, propranolol-*d*_7_, temazepam-*d*_5_ and diazepam-*d*_5_ were ordered from Sigma-Aldrich. Sulfamethazine-*d*_*4*_, nifedipine-*d*_*4*_, metoprolol-*d*_*7*_, trimethoprim-*d*_3_ and warfarin-*d*_5_ were ordered from QMX Laboratories (Essex, UK). The propranolol biotransformation products; 4-hydroxypropranolol, 4-hydroxypropranolol sulphate and 4-hydroxypropranolol glucuronide were sourced from Santa Cruz Biotechnology (Heidelberg, Germany). All pharmaceuticals were of a purity of ≥97%. Analytical grade salts (>99%) including sodium hydrogen carbonate, magnesium sulphate, calcium sulphate, potassium chloride were purchased from Sigma. Ultra-pure water was obtained from a Millipore Milli-Q water purification system with a specific resistance of 18.2 MΩ cm or greater (Millipore, Bedford, MA, USA).

### 2.2 Sample collection and culture maintenance

*Gammarus pulex* were collected by kick-sampling from the River Cray, South-East London, UK, 51°23′09.5″N 0°06′32.4″E. This site was previously shown to have low pharmaceutical contamination in both collected surface water and animal samples (Miller et al., 2015). The populations were transported to the laboratory in 500 mL Nalgene™ flasks filled with surface water from the sample collection site. Populations were rinsed with artificial freshwater (AFW) and then acclimatised to laboratory conditions (as specified below) for a minimum of 7 days before any exposure experiments were performed. AFW was prepared from 1.15 mM of NaHCO_3_, 0.50 mM MgSO_4_, 0.44 mM CaSO_4_ and 0.05 mM of KCl dissolved in 20 L of ultra-pure water. This water was subsequently aerated for several hours to remove dissolved carbonic acid and maximise the dissolved oxygen concentrations. Each culture tank (n = 8) was filled with 2.5 L of AFW and animals were fed with either alder or horse chestnut leaves obtained from the sampling site and conditioned by submersion in surface water for two days prior to use.

### 2.3 Toxicokinetic exposure and conditions

Toxicokinetic experiments were performed separately for each pharmaceutical for a total of 96 h which included a 48 h uptake phase followed by a 48 h depuration period as per (Miller et al., 2016). Individual adult organisms (n = 25), both male and female (>5 mg wet weight) were placed in Pyrex glass beakers. *G. pulex* were carefully transferred to beakers using blunt forceps to avoid any harm to the organisms before exposure. Each beaker contained 25 organisms in 200 mL of exposure media (AFW and test compound). *G. pulex* were exposed to individual pharmaceuticals at a concentration of 1 μg L^−1^, except for propranolol and warfarin which were exposed at 10 μg L^−1^. All exposure media contained <0.001% of organic solvent (methanol). A total of 300 organisms were used per compound exposure and were sampled for n = 3 per time-point. Samples were taken at 6, 24, 48 and 96 h across the toxicokinetic experiments. Negative control exposures were set up and also sampled at the 96 h time interval. These were subsequently analysed for background contamination. The exposure media was replaced daily and water samples (n = 3) were analysed at 0 and 24 h after exposure to ensure concentrations of the compounds remained constant in the AFW. Each animal specimen was rinsed with ultra-pure water and then frozen at −20 °C. The light cycle was 12 h light followed by 12 h dark without a dusk/dawn transition period. All experiments were performed in a temperature controlled room at 15 °C (±2 °C) and water pH was also monitored across each experiment and measured at an average of 8.19 ± 0.05.

### 2.4 Sample preparation

Water samples collected from exposure experiments were filtered using Whatman filters (0.2 μM) and directly injected onto the LC-MS/MS for analysis. For *G. pulex*, A mass of 50 mg of lyophilised and ground material was extracted by glass bead pulverisation in 5 mL of acetonitrile and diluted with 100 mL of ammonium acetate buffer (10 mM) for loading onto Waters HLB solid phase extraction (SPE) cartridges (6 cc, 200 mg sorbent). After loading, samples were eluted using a mixture of ethyl acetate:acetone (50:50 v/v) which was subsequently evaporated under nitrogen (99% purity) and reconstituted in 250 μL of starting LC mobile phase (90:10 v/v, 10 mM ammonium acetate buffer: acetonitrile).

### 2.5 Instrumental analysis

Full instrumental conditions were used as per (Miller et al., 2015). Briefly, separations were performed on an Agilent HP1100 LC system configured with a Waters SunFire reversed-phase C_18_ column (150 mm × 2.1 mm, 2.5 μM particle size). Following injection (20 μL), elution followed a gradient profile (totalling 75 min including a 12.5-min re-equilibration time) using a mixture of acetonitrile and ultra-pure water with 10 mM ammonium acetate. Detection was performed using a Waters Quattro triple quadrupole mass spectrometer with electrospray ionisation operated in positive and negative polarity switching mode. Quantification of analytes was performed using 3-point internal standard matrix-matched calibration using the peak area ratio of non-labelled analyte (precursor range: 50–300 ng g^−1^, biotransformation product range: 50–150 ng g^−1^) to the stable isotope labelled internal standard (SIL-IS) (100 ng g^−1^). The exception to this was in the temazepam exposure where quantification was performed against a SIL-IS calibrant concentration (100 ng g^−1^) by comparing the response ratio of SIL-IS:unlabelled temazepam. The compounds (4-hydroxypropranolol sulphate and carbamazepine-10,11-epoxide) were quantified using 3-point external matrix-matched calibration curves due to unavailability of SIL-IS standards.

### 2.6 Modelling bioconcentration factors

Parameter estimation of uptake rate constant (*k*_1_) and depuration rate constant (*k*_2_) was performed using a curve fitting algorithm via Minitab statistical software (Minitab Ltd., Coventry UK) and as outlined in the OECD 305 Fish Bioconcentration Guidelines (OECD). Full details of parameter estimation can be found in (Miller et al., 2016). Herein, the authors define BCF_total_ as the summation of the BCF of both parent/precursor and biotransformation products (i.e. the total body burden). Whereas, BCF_parent_ will be used to describe the BCF determined for the parent compound alone.

## 3 Results and discussion

### 3.1 Analytical performance and minimised test design evaluation

The previously described analytical method required a sample mass of 100 mg dw, which corresponded to approximately 40 animals per measurement (Miller et al., 2015). This presented feasibility issues for sampling, maintaining cultures and the scale of exposure experiments. To mitigate this, 50 mg was used instead and the reconstitution volume of solvent (post-SPE) was also scaled down (to 250 μL) to maintain sensitivity. Analytical method performance was reassessed for 10 pharmaceuticals (See SI, Fig. S1 and Table S1). In general, imprecision increased with decreasing sample mass extracted. The use of SIL-IS has been shown to offer improved precision during analytical method development (Stokvis et al., 2005). The corresponding SIL-IS for each analyte here also resulted in markedly improved precision for all compounds with % RSDs ranging from 1 to 11%. Linearity also improved using SIL-IS in comparison to matrix-matched calibration curves (*R*^*2*^ ≥0.9897). Therefore, for precise analysis of biota like *G. pulex* for trace pharmaceutical residue determination, it is recommended that SIL-IS be used with LC-MS/MS to overcome precision problems relating to the limited sample mass available and to enable the number of specimens to be minimised.

In addition to the reduction in the number of organisms, a reduction in the number of sampling time intervals was also considered. Time points were selected at 6, 24, 48 and 96 h so that the uptake phase had three time points and the elimination phase contained two intervals. The additional time point in the uptake phase was selected so that any losses in *k*_1_ constancy could be highlighted as identified previously (Miller et al., 2016). The potential limitation when using a small number of time intervals is that the data may not reflect reliably the ability of a compound to concentrate as modelling is limited to a few data points. The OECD 305 guidelines do propose a minimised test design with two time intervals in the uptake and elimination phase, respectively (e.g. day 14, 28, 35 & 42). This minimised study design was evaluated independently in the literature and it was concluded that the test was valuable and offered reliable BCF estimation for regulatory purposes (Carter et al., 2014). Springer et al. proposed a second minimised test design involving only two sampling time intervals in a 14 d depuration period (Springer et al., 2008). They also found that minimised test designs were a viable alternative to a full study design. Given that our experiments here focused on a non-standard invertebrate species and much shorter uptake and depuration phases, re-evaluation of the uptake and elimination data from (Miller et al., 2016) was performed to assess the suitability of a minimised test design for simultaneous and sequential methods of BCF estimation in *G. pulex* (Table S3). An ANOVA was performed for each method of estimation and resulted in *p*-values of 0.95 for comparison of simultaneous BCF_total_, 0.43 for sequential BCF_total_ and 0.45 for sequential BCF_total_ determined using linear regression in the elimination phase data. All three *p*-values were >0.05 indicating that there were no statistically significant differences between the BCFs estimated by the full study design nor the minimised design. Therefore, going forward all non-radiolabelled exposures were performed using the minimised design.

### 3.2 Toxicokinetic modelling of eight selected pharmaceuticals

Aqueous pharmaceutical concentrations remained stable across the exposure period for carbamazepine, diazepam, temazepam, sulfamethazine, metoprolol and trimethoprim (Table 1). Propranolol showed stable aqueous concentrations over the first 24 h, but declined by 29% to an average of 6.41 μg L^−1^ thereafter. Nifedipine showed an average decrease of 39% across both days of the uptake phase. It is possible that sorption was the cause in the reduction of nifedipine in the exposure media or could also be attributed to other transformation processes.

**Table 1:** Pharmaceutical concentrations in exposure media during the uptake phase. 24 h and 48 h represent the concentration after 24 h of exposure with solutions used on either Day 1 or Day 2.

Compound	Concentration (μg L^−1^)						
	Day 1 (n = 3)	SD	24 h (n = 3)	SD	Day 2 (n = 2)	48 h (n = 3)	SD
Carbamazepine	1.12	0.03	1.01	0.05	1.08	1.07	0.02
Diazepam	1.02	0.03	0.86	0.05	1.04	0.89	0.11
Temazepam	0.96	0.03	0.85	0.03	1.17	1.03	0
Nifedipine	0.94	0.09	0.59	0.06	0.82	0.49	0.18
Sulfamethazine	1.06	0.07	0.99	0.17	0.89	0.71	0.15
Trimethoprim	1.09	0.04	1.01	0.11	0.99	0.97	0.04
Metoprolol	0.77	0.18	0.72	0.15	0.86	0.87	0.21
Propranolol	9.22	0.5	8.92	0.29	8.98	6.41^a^	–

Day 1- initial pharmaceutical concentration on day 1.

Day 2- initial pharmaceutical concentration on day 2.

a n = 2

For pharmaceuticals in *G. pulex*, maximal concentrations after the uptake phase were observed for propranolol and likely due to its higher exposure concentration at 10 μg L^−1^ (due to a relatively high LOQ value (61 ng g^−1^ dw). In this exposure, internal concentrations reached 519 ± 143 ng g^−1^ with a mean value of 210 ± 9 ng g^−1^ at the end of the uptake period. Warfarin also showed relatively higher concentrations in *G. pulex* which again was likely explained by the higher exposure concentration. In contrast to the other studied compounds, the data for warfarin suggested that it did not reach/approach steady state in the uptake period as no plateau was observed in the toxicokinetic profile. The remaining pharmaceuticals carbamazepine, diazepam, metoprolol, nifedipine, trimethoprim and temazepam exposed at 1 μg L^−1^ showed internal concentrations of ≤51 ng g^−1^ at the end of the uptake phase. These internal concentrations showed rapid elimination and were reduced to ≤ LOQ/LOD. The rapid turnover of all pharmaceuticals suggested that bioaccumulation could be less relevant for these types of ionisable compounds. BCFs were generated using both simultaneous and sequential modelling presented in Table 2. The BCF_parent_ generated for each compound was in the order of trimethoprim and nifedipine < metoprolol < warfarin < carbamazepine < propranolol < temazepam < diazepam. The highest BCF_parent_ generated by the simultaneous method was 41 L kg^−1^ for diazepam and the lowest estimation was 16 L kg^−1^ for both trimethoprim and nifedipine. These values remain significantly lower than any regulatory threshold to be considered bioaccumulative or very bioaccumulative (European Commission, 2006). The BCF_parent_ values for propranolol and metoprolol were also compared with the BCF_total_ generated previously (Miller et al., 2016). Propranolol BCF_total_ was estimated to be 32 L kg^−1^ and metoprolol BCF_total_ was 16 L kg^−1^, which showed very good agreement with the BCF_parent_ values of 28 and 17 determined by LC-MS/MS here. Sulfamethazine was not detected in any sample and therefore indicated that no accumulation in *G. pulex* had occurred. Interestingly, exposure to nifedipine (log*P* = 3.45) resulted in a low BCF despite it being less polar and was similar to that of trimethoprim (log*P* = 1.12). This further suggested that log*P* was not a reliable indicator for BCF of pharmaceuticals and the degree of ionisation may also play an important role in uptake and bioconcentration mechanisms. Uptake models have usually been based on neutral organic micropollutants and is the reason that log*P* can be a good indicator of bioconcentration for these compounds especially when log*P* is < 6. The log*P*, log*D* and predominant form of each pharmaceutical exposed to *G. pulex* is shown in Table S4. Temazepam, diazepam and carbamazepine remain neutral, but their respective BCF_parent_ do not follow any specific trend when directly compared to their log*P*. However, the selection of pharmaceuticals here is limited and therefore discernible trends may not be apparent. A plot of log*D*/*P* versus estimated BCF showed that there were no identifiable trends (Fig. S2). Consideration of the complexity of biological systems and the unique characteristics of pharmaceuticals, it may be expected that uptake and bioconcentration is a much more complex process than can be predicted by a single or small number of physicochemical properties. Many factors have been proposed and reported to influence the bioconcentration of pharmaceuticals including hydrophobicity, biotransformation, bioavailability, environmental exposure scenarios, and carrier mediated uptake (Barron, 1990). A particularly important factor is the environmental exposure as the complexity of environmental matrices on BCFs may well increase or decrease predicted BCFs or measured BCFs from *in vivo* laboratory exposures. Surface water temperature, pH, concentrations in sediment and the composition of other components in the surface water (e.g. complex mixtures including other pharmaceuticals, colloids, surfactants, organic chemicals etc.) may directly or indirectly affect the accumulation potential of a compound (Brown et al., 2007, Chen et al., 2017, Nichols et al., 2015).

**Table 2:** Determination of BCFs using either simultaneous or sequential parametrisation of *k*_1_ and *k*_2_.

Compound	Simultaneous						Sequential^a^						Sequential^b^					
	*k*_1_	SE	*k*_2_	SE	*p*-value	BCF_parent_	*k*_1_	SE	*k*_2_	SE	*p*-value	BCF_parent_	*k*_1_	SE	*k*_2_	*r*^2^	*p*-value	BCF_parent_
Carbamazepine	0.5307	0.115	0.0214	0.008	0.049	25	0.4418	0.054	0.014	0.007	0.07	32	0.4643	0.056	0.0158	0.534	0.08	29
Diazepam	9.5942	2.126	0.2349	0.058	0.028	41	2.6469	0.265	0.046	0.011	0	58	3.0917	0.28	0.0582	0.953	0.001	53
Temazepam	4.3326	0.556	0.1186	0.018	0.131	37	2.8514	0.116	0.0702	0.027	0.022	41	2.751	0.117	0.0669	0.9965	0.016	41
Trimethoprim	2.325	0.916	0.1488	0.066	0.376	16	0.8451	0.105	0.0402	0.017	0.066	21	0.837	0.105	0.0396	0.8468	0.064	21
Nifedipine	4677.31	n/a	295.66	n/a	n/a	16	0.7802	0.146	0.0248	0.001	0	31	0.7802	0.146	0.0248	0.9967	0	31
Warfarin^c^	0.2242	0.022	0.0119	0.003	0.043	19	0.2091	0.012	0.0096	0.003	0.066	22	0.2118	0.012	0.01	0.6989	0.072	21
Metoprolol	0.4413	0.135	0.0259	0.015	0.005	17	0.2788	0.046	0.0071	0.006	0.005	39	0.2855	0.047	0.0078	0.39	0.006	37
Propranolol^c^	6.596	5.797	0.2348	0.221	0.027	28	0.8059	0.181	0.0111	0.002	0.006	73	0.8116	0.182	0.0113	0.8543	0.006	72

SE – Standard Error.

a Determined using Levenberg-Marquardt algorithm.

b Determined using linear regression on elimination data.

c Exposed at 10 μg L^−1^.

Few data or reported studies exist on the uptake of pharmaceuticals in invertebrates. However, comparison of the BCF_parent_ estimated here showed good agreement with a study on *G. pulex* by Meredith-Williams et al. for two compounds, carbamazepine and diazepam, (Meredith-Williams et al., 2012). Sordet et al., reported carbamazepine BCF of 5 L kg^−1^ in *Gammarus fossarum* which was ∼5-fold lower than the BCF determined here (Sordet et al., 2016). Another study also reported a similar BCF of 51 L kg^−1^ for diazepam in a marine mussel, *Mytilus galloprovincialis* (E. Gomez et al., 2012). In *Daphnia magna*, BCFs of between 18 and 83 L kg^−1^ for propranolol were reported, but the estimated value varied with exposure concentration (Ding et al., 2016). A review of fish data generally revealed low level bioconcentration of the selected pharmaceuticals herein. For example, diazepam BCF in fish (*Ictalurus punctatus*) ranged from 2 to 146 L kg^−1^ depending on the tissue type (Overturf et al., 2016); sulfamethazine BCF in sturgeons was shown to be 1 L kg^−1^ (Hou et al., 2003); carbamazepine BCF in two species of fish (*Pimephales notatus* and *I. punctatus*) was <7 L kg^−1^ depending on tissue (Garcia et al., 2012); and propranolol was also estimated to have a BCF of <1 L kg^−1^ in *Oncorhynchus mykiss* and *I. punctatus* (C. F. Gomez et al., 2010). These data further support the results herein and indicate that the selected pharmaceuticals are likely to have a low potential for bioconcentration in aquatic species.

**Fig. 1:**
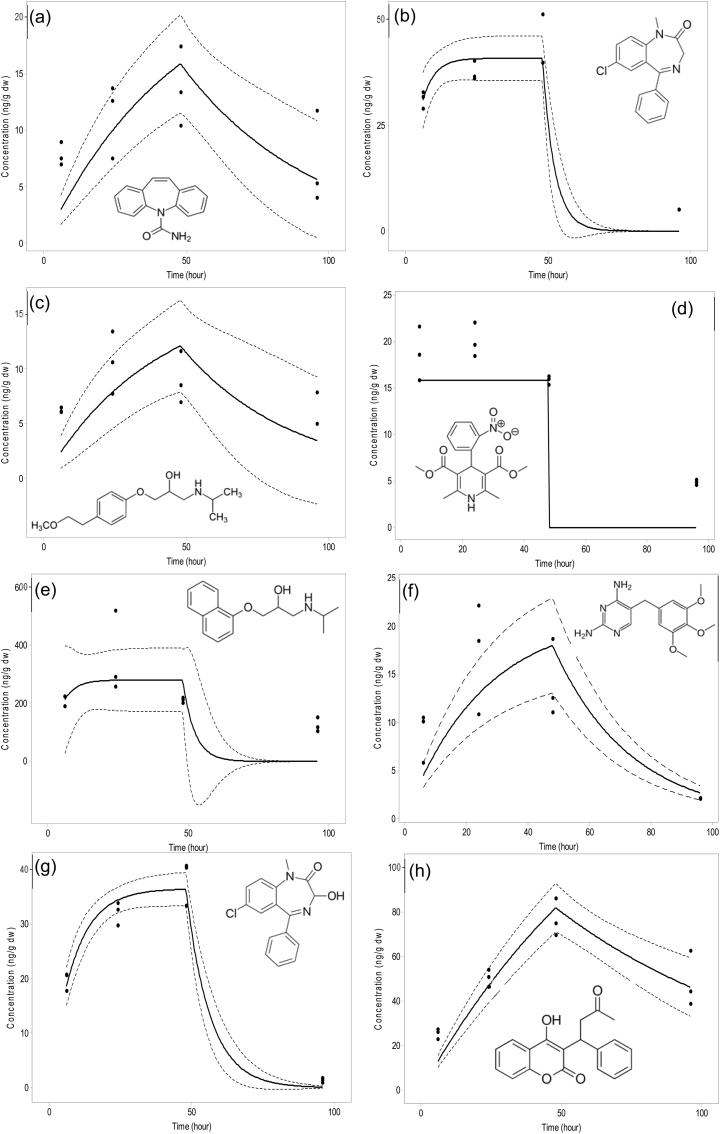
Toxicokinetic profiles of selected pharmaceuticals in *G. pulex* measured using LC-MS/MS. Solid line represents model fit, dashed lines represent 95% confidence interval. (a) carbamazepine, (b) diazepam, (c) metoprolol, (d) nifedipine, (e) propranolol, (f) trimethoprim, (g) temazepam and (h) warfarin.

**Fig. 2:**
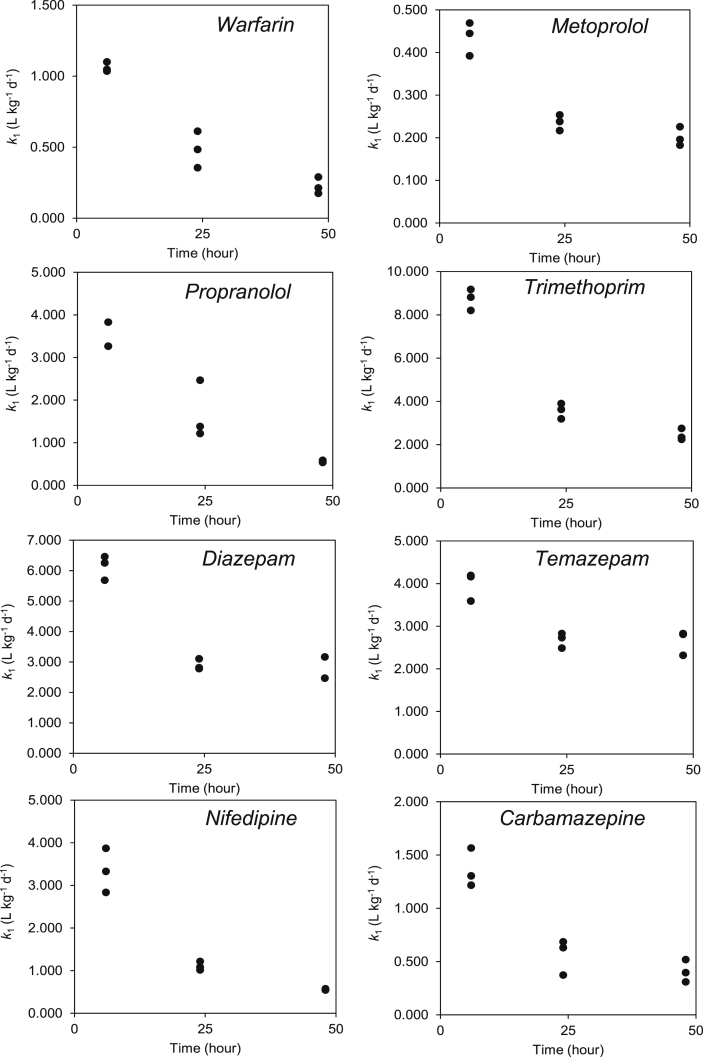
Plots of *k*_1_ over time for the selected eight pharmaceuticals.

The model fits of simultaneous and sequential BCF_parent_ estimation showed a significant lack-of-fit for several pharmaceuticals (Fig. 1). Lack-of-fits may arise from a large scatter in the data. However, the advantage of pooling organisms through LC-MS/MS measurements is that it reduces scatter of measured internal concentrations from single organisms that arises from inter-individual variability. The lack-of-fit observed seemed to arise from the uptake phase data and therefore the rate constant was estimated over each time interval during the uptake period. This revealed that there was a decreasing trend in the *k*_1_ rate constant which again did not obey the model's assumptions. Lack-of-fits were observed in the simultaneous method of estimation for five compounds (carbamazepine, propranolol, metoprolol, warfarin and diazepam) with two compounds showing no significant lack-of-fits (temazepam and trimethoprim). Lack-of-fit for the final compound nifedipine was not possible to estimate due to line that was fit (Fig. 1(d)). As several significant lack-of-fits were observed, the *k*_1_ rate constant was re-calculated over the time intervals for the uptake phase data (Fig. 2). Again, *k*_1_ was observed to decrease over time. These decreases again are potentially the cause of the lack-of-fits, as suggested previously (Miller et al., 2016). The reason for the decrease in *k*_1_ over time could be attributed to either sorption on to the cuticle of the animal during the uptake phase, toxicodynamic effects where the pharmaceuticals are reducing the ability of the organism to eliminate the compound or growth dilution of the animal. However, preliminary evidence in (Miller et al., 2016) and the larger decreases in *k*_1_ at the earlier time interval (this study) suggest that sorption might be a significant cause of the decreases in *k*_1_ over time. In the cases of warfarin, carbamazepine, diazepam and temazepam an apparent plateau had been reached indicating *k*_1_ constancy and it was possible to use this value to estimate the BCF_parent_. The average of the *k*_1_ over the 48 h time interval for these compounds resulted in a BCF_parent_ of 20, 26, 40 and 48 L kg^−1^ for warfarin, carbamazepine, temazepam and diazepam, respectively. These values showed good agreement between the BCFs generated by the simultaneous method indicating this could provide an alternative method for BCF estimation when *k*_1_ values are found to decrease over time. However, it is also possible that steady-state measurements are more appropriate over kinetic measurements when estimating pharmaceutical BCFs. Behaviour of pharmaceuticals is also different to more traditional organic pollutants (PAHs, PCBs, etc.), it appears they often reach steady-state within a relatively short timeframe (∼2–3 days) (Meredith-Williams et al., 2012, Miller et al., 2016). Thus, determination of BCFs using kinetic parameterisation offers no advantage over the time requirements for steady-state BCF estimates. Furthermore, steady-state measurements would also be less resource intensive as they do not require a depuration period. In addition, the inconsistencies of kinetic data as demonstrated here and in (Miller et al., 2016) might not provide a reliable estimate of BCFs.

### 3.3 Biotransformation of carbamazepine, diazepam and propranolol by G. pulex

In addition to their medium to low log*P*, pharmaceuticals are designed to be metabolised and excreted to minimise accumulation (Kumar and Surapaneni, 2001). As the data suggests, factors other than hydrophobicity may be important in accumulation, such as biotransformation, which could also partly explain the variability in BCFs. Authors have demonstrated that *G. pulex* are able to metabolise a range of organic micropollutants (Ashauer et al., 2012, Jeon et al., 2013b, Rösch et al., 2016). Conservation of cytochrome P450 enzymes has also been observed in invertebrates (Snyder, 2000) and pharmaceuticals have been shown to undergo oxidative and conjugation reactions (Jeon et al., 2013b). As the targeted LC-MS/MS method only determines the amount of parent compound, the BCF_parent_ values presented above (Table 2) do not take into account the accumulation of any biotransformation products. The biotransformation product half-lives in the body can be longer or shorter than the parent compound as they are modified by biotransformation processes and potentially may lead to increased or decreased accumulation (Jeon et al., 2013a) and thus will not be accounted for by estimations of BCF_parent_.

Selected pharmaceuticals with readily available biotransformation product reference standards (propranolol, carbamazepine and diazepam) were used in biotransformation studies and several transformation products were targeted in the analytical method including 4-hydroxypropranolol, 4-hydroxypropranolol sulphate, 4-hydroxypropranolol glucuronide, carbamazepine-10,11-epoxide, oxazepam and nordiazepam and temazepam, the latter of which was already included in analytical method. A matrix effect and recovery experiment was performed for these additional analytes before exposures were performed (SI Table S5). Unfortunately, poor stability in solution made the matrix effect or recovery assessment for 4-hydroxypropranolol impossible. This has been reported previously (Pritchard et al., 1979) and samples required additives (sodium metabisulfite and sodium bisulfite) to maintain stability. Overall, matrix effects were relatively minor for most biotransformation products ranging from 4% suppression to 9% enhancement. However the exception was nordiazepam which showed 31% signal suppression. biotransformation products showed acceptable absolute recoveries ranging 82–103% (≤13% RSD) with the exception of 4-hydroxypropranolol sulphate (34% recovery and 20% RSD) and 4-hydroxypropranolol glucuronide (no recovery). The lower recoveries and precision of these polar conjugates are likely to arise from poor affinity to the SPE sorbents (HLB) used during sample preparation. The lower recoveries and precision of these polar conjugates are likely to arise from poorer affinity to these SPE sorbents. The use of alternative chemistries including mixed-mode ion exchangers or dipole bearing polymers may improve the selectivity for such polar compounds.

**Fig. 3:**
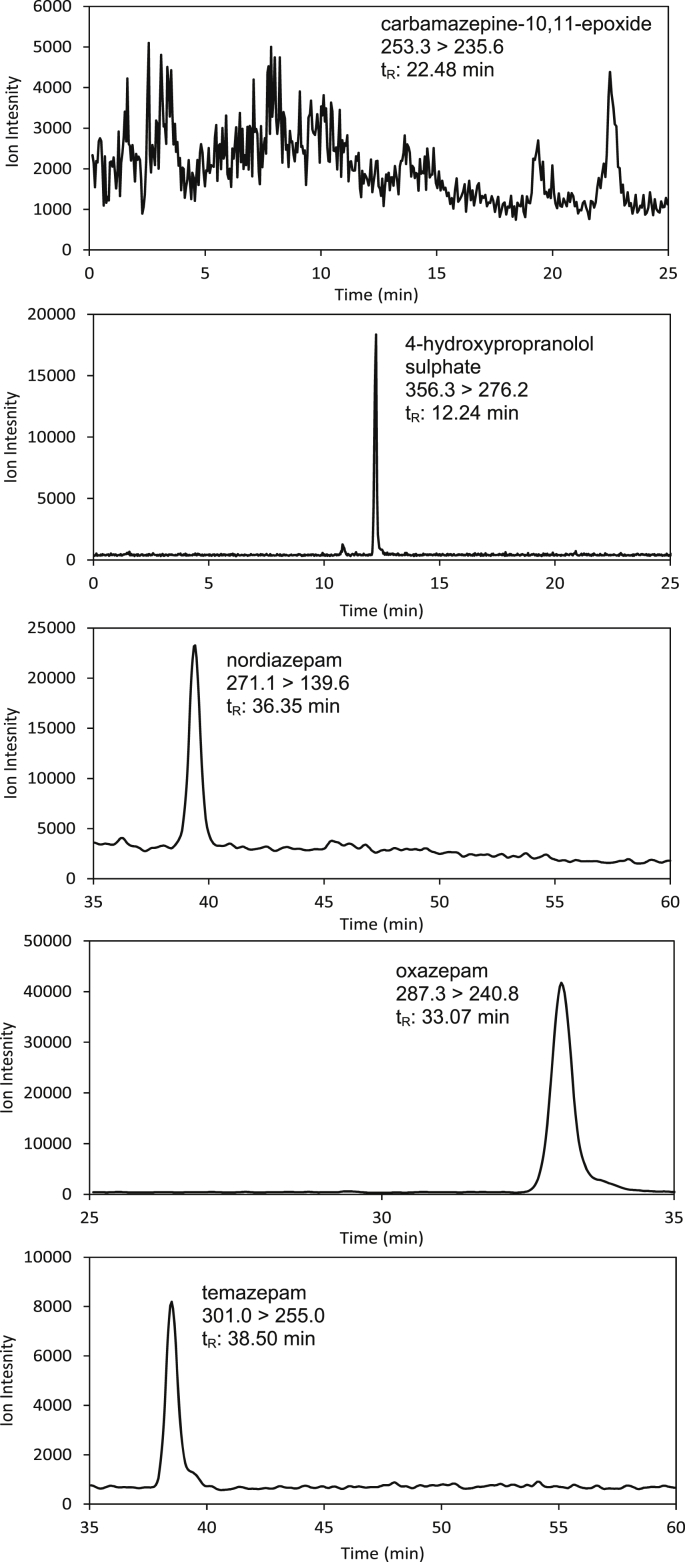
SRM transitions for the detected biotransformed products during toxicokinetic exposures.

Carbamazepine exposures resulted in the detection of carbamazepine-10,11-epoxide at 24 h and 48 h in *G. pulex*. Control organisms (exposed to AFW only) also showed no detectable peaks for carbamazepine-10,11-epoxide. This was identified by a single *m*/*z* transition (253 → 235) and chromatographic retention time (within 0.4%). Unfortunately, carbamazepine-10,11-epoxide was not quantifiable as signals were below a signal to noise ratio of 10:1 (Fig. 3). Carbamazepine-10,11-epoxide was not detected at 96 h suggesting that the biotransformation product may have been eliminated from the organism by this point. Elimination may either be via excretion or further biotransformation, e.g. carbamazepine-10,11–diol is excreted in its free form or as a glucuronic acid conjugate in humans (Kudriakova et al., 1992). However, limited data is available for biotransformation of xenobiotics in invertebrates. A previous study identified that carbamazepine was converted to carbamazepine-10,11-epoxide and was the main metabolic pathway in the mussel, *Mytilus galloprovincialis* (Boillot et al., 2015). Fish exposed to carbamazepine have also shown the presence of two carbamazepine biotransformation products, carbamazepine-10,11-epoxide and 2-hydroxycarbamazepine (Boillot et al., 2015).

**Fig. 4:**
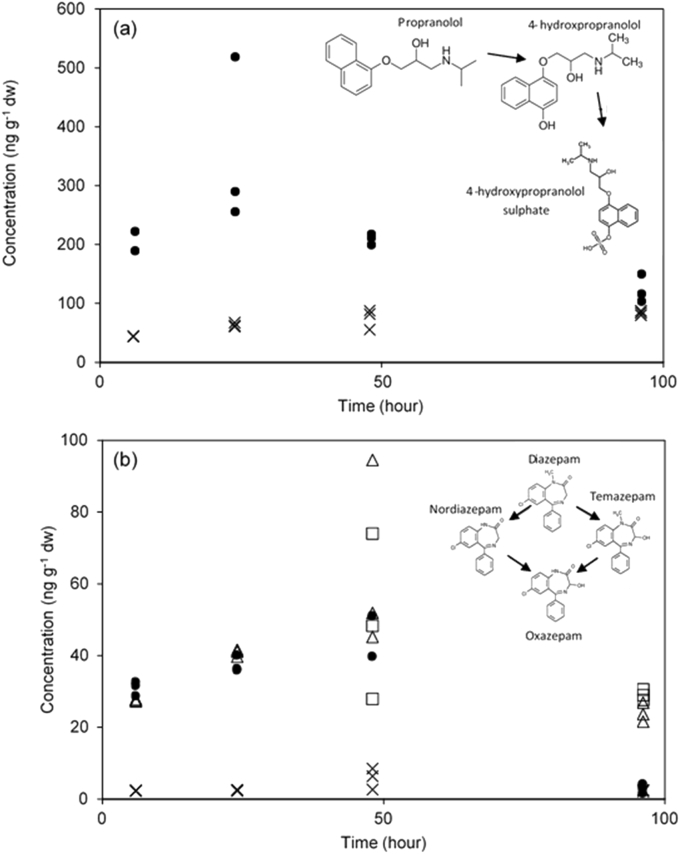
Determination of biotransformation products (a) Concentration-time profile for propranolol (solid circles) and 4-hydroxypropranolol sulphate (crosses) in *G. pulex*. (b) Concentration-time profile for diazepam (solid circles), nordiazepam (triangles), oxazepam (squares) and temazepam (crosses) in *G*. *pulex*.

For propranolol exposures, 4-hydroxypropranolol sulphate was confirmed with a single peak transition (356.3 → 276.2) and chromatographic retention time (within 0.25%) (Fig. 3). HP-SULPH reached a mean concentration of 75 ± 17 ng g^−1^ dw by the end of the uptake phase (Fig. 4(a)). The elimination phase showed no decreases in the concentration 4-hydroxypropranolol sulphate which was measured at a mean concentration of 84 ± 4 ng g^−1^ dw at 96 h. Determination of the parent compound propranolol showed a peak at the 24 h time interval which decreased by 48 h. Decreases of internal concentrations during the uptake phase are indicative of active metabolic pathways (Crookes and Brooke, 2011). To the authors’ knowledge, no propranolol biotransformation products have previously been specifically identified using LC-MS/MS in either fish or invertebrates. However, from the induced P450 activity of trout *in vivo* and *in vitro* propranolol was suggested to likely induce its metabolism and it was hypothesised that biotransformation products may be identified (Bartram et al., 2012). More recently trout gill cells have been shown to be capable of propranolol transport and biotransformation (Stott et al., 2015), and in carp a range of biotransformation products were tentatively identified *in vivo* using UV methods (Ding et al., 2015). Furthermore, sulphate conjugates of pharmaceuticals have not been previously detected in invertebrates. However, previous works have detected sulphate and glucose conjugates of pyrene in *Daphnia magna* (Ikenaka et al., 2006, Ikenaka et al., 2007) and sulphate conjugates of aldicarb, carbaryl and dichlorophenols in *G. pulex* (Ashauer et al., 2012). For biocides (algicides), glutathione conjugates have been detected in *G. pulex* and *D. magna* (Jeon et al., 2013b). Finally, *G. pulex* have also been shown to biotransform azole fungicides into sulphate, glutathione and glucose-sulphate conjugates (Rösch et al., 2016). Human trials show that the major biotransformation products of propranolol were HP and napthoxylactic acid (Walle and Gaffney, 1972). The relative importance of this sulphate conjugation pathway in *G. pulex* is not known as the napthoxylactic acid and the glucuronide conjugate could not be determined. However, some authors have suggested that sulphate and glucoside conjugation is the major metabolic process in invertebrates for the metabolism of aryl group containing compounds such as propranolol (Ikenaka et al., 2007, Livingstone, 1998).

The final exposure was performed with diazepam and all three selected biotransformation products were detected and quantified (Fig. 4(b)). Nordiazepam showed concentrations that reached a mean concentration of 64 ± 27 ng g^−1^ dw in contrast to temazepam that reached a maximum mean concentration of 6 ± 3 ng g^−1^ dw at 48 h. The 10-fold difference in concentrations suggested that the biotransformation of diazepam to nordiazepam is the major metabolic pathway in contrast to the conversion of diazepam to temazepam. This agrees with mammalian data that shows the demethylation of diazepam to nordiazepam is the primary metabolic pathway (Umezawa et al., 2008). Temazepam was not detectable by the 96 h time interval, suggesting this compound had been either excreted or further biotransformed to oxazepam. The *k*_2_ was determined at 0.0194 d^−1^ which is 3-fold lower than the *k*_2_ of diazepam. The estimated half-lives of diazepam and nordiazepam were 12 h and 36 h, respectively. The difference observed is also in agreement with the reported half-lives, as nordiazepam has a longer half-life (50–120 h) than its parent compound diazepam (44 h) (Umezawa et al., 2008). The *k*_2_ of temazepam was estimated at 0.016 d^−1^, approximately 4-fold lower than the *k*_2_ of temazepam when exposed to *G. pulex* as a parent compound. The lower *k*_2_ may be explained by the apparent preferential metabolism of diazepam to nordiazepam. Thus, enzymes involved in the temazepam pathway may be less active.

The final biotransformation product oxazepam was not detectable until 48 h reaching a mean concentration of 50 ± 23 ng g^−1^ dw. The biotransformation of diazepam to either nordiazepam or temazepam and further conversion to oxazepam would be rate liming steps leading to the apparent lag phase in the detection of oxazepam. The *k*_2_ estimated for oxazepam was 0.009 d^−1^, with mean internal concentrations reduced by 21 ng g^−1^ dw over the 48 h depuration period. The half-life determined for oxazepam was 70 h which is much greater than the reported single or multiple dose half-life in humans (9–11.6 h) (Greenblatt, 1981). The difference in half-lives could be due to the continued conversion of nordiazepam or temazepam to oxazepam after the uptake phase ended giving an apparent longer half-life. Furthermore, oxazepam is primarily excreted by conjugation with glucuronide moieties indicating that *G. pulex* may not readily metabolise oxazepam as well as in humans. The degree of accumulation of each biotransformation product is in agreement with Overturf et al., whom generally found higher concentrations of nordiazepam and oxazepam in comparison to temazepam dependent on the tissue type (Overturf et al., 2016).

Metabolite enrichment factors (MEFs) can be determined for biotransformation products (Ashauer et al., 2012). The MEFs can be likened to a ‘pseudo-BCF’ and further indicate that these biotransformation products were not accumulative. However, nordiazepam and oxazepam reached higher internal concentrations relative to the parent compound diazepam. The BCF of oxazepam has been reported at 22 L kg^−1^ in *G. fossarum* indicating that this compound is not accumulative (Sordet et al., 2016). Furthermore, the summation of the BCF_parent_ and MEFs can give a BCF that would be comparable to those determined by total radioactivity counts (i.e. BCF_total_) (Ashauer et al., 2012). For example, the BCF_parent_ of diazepam ranged from 41 to 58 L kg^−1^ whereas summation of the diazepam BCF and the MEFs would give a BCF_total_ estimate of 165 L kg^−1^. However, it should be considered that targeted methods will likely only show a small window of the biotransformation pathways involved with xenobiotic detoxification (as in this work). Secondly, targeted methods may not focus on the important biotransformation products in terms of accumulation, toxicity and elimination. Therefore, untargeted analytical techniques such as high resolution mass spectrometry (HRMS) could potentially offer a much greater insight into biotransformation pathways involved with xenobiotic detoxification or identification of new compounds (Ashauer et al., 2012, Munro et al., 2015). However, the quantitative application of LC-HRMS to toxicokinetic profiling needs to be considered carefully, especially where reference materials for biotransformation products are not available as discussed previously (Jeon et al., 2013a, Rösch et al., 2016). Furthermore, studies that only monitor the parent compound by non-specific methods should be cautious when reporting BCFs, especially if there is a high potential for biotransformation. Comparison of the total BCF (BCF_parent_ + MEF) to BCFs determined by LSC was not possible here. However, Ashauer et al. (2012) reported that comparison of total BAF and BAF by LSC gave values that were within a single order of magnitude. However, the differentiation between biotransformation products and their respective parents gave better accuracy in parameter estimates (*k*_1_/*k*_2_) compared to radioactivity measurements (Ashauer et al., 2012). The reason for this is that radioactivity measurements can over or underestimate elimination if biotransformation is not taken into account. As a final consideration, the data presented show that at environmentally relevant exposure concentrations, pharmaceuticals remain at very low level concentrations. Furthermore, for the selected compounds herein, they do not show any significant accumulation which has also been evidenced in the literature by several authors (Boillot et al., 2015, Meredith-Williams et al., 2012, Miller et al., 2016, Paterson and Metcalfe, 2008, Sordet et al., 2016). Thus, biotransformation studies will be key in highlighting the behaviour of these contaminants inside the animal and reveal the role of metabolic clearance for regulating accumulation. In addition, whilst the accumulation potential of pharmaceuticals is low, it must now be considered how these innately low level concentrations of precursor and biotransformed products will affect the organisms that are exposed to them. Thus, future work should aim to link accumulation data to effect data for more comprehensive understanding of the potential for adverse outcomes of these emerging contaminants.

## 4 Conclusions

As an alternative to traditional LSC approaches, LC-MS/MS was shown as a suitable technique for the measurement of uptake and elimination kinetics. The simultaneous BCF estimates ranged from 16 to 41 L kg^−1^ for eight compounds (diazepam, temazepam, nifedipine, propranolol, metoprolol, carbamazepine, warfarin and trimethoprim) using the simultaneous model method. Sequential parameterisation resulted in BCFs of 21–72 L kg^−1^ showing overestimates compared to the simultaneous method. Sulfamethazine showed no bioconcentration in the animals, as no peaks were detected upon exposure. Models were shown to have significant lack-of-fits for six of the eight pharmaceuticals. The lack-of-fits also coincided with decreases in the uptake rate constant over time suggesting that poor model fits may have resulted from this trend. No trends in bioconcentration were observed with log*D* or log*P*, suggesting factors other than compound hydrophobicity were important in bioconcentration. The role of metabolism was investigated for three selected pharmaceuticals (carbamazepine, propranolol and diazepam). *G. pulex* were shown to metabolise all three pharmaceuticals into several different biotransformation products, indicating the conservation of cytochrome P450 enzymes in this species. Furthermore, detection of 4-hydroxpropranolol sulphate indicates the presence of transferases. The ability of *G. pulex* to readily metabolise these xenobiotics may explain, in part, the relatively low BCFs determined for pharmaceuticals in this work and the literature. Biotransformation pathways and products were found to be the same between vertebrate data. However, differences between half-lives were observed for the benzodiazepine compounds (diazepam = 12 h, nordiazepam = 36 h) suggesting that rates of metabolism and elimination are different. Whilst, kinetics may differ, the same metabolic pathways involved in elimination mean that human pharmacokinetic data is valuable for consideration of pharmaceuticals in environmental risk assessment. Analytical methods that only target and determine the parent compound in toxicokinetic studies do not measure a BCF_total_. As MEFs for the diazepam biotransformation products, nordiazepam and oxazepam, showed that some compounds may be more accumulative than the parent and could potentially be more toxic. Therefore, it is advisable that targeted MS methods account for biotransformed products when estimating BCFs.
