# Supplementary material for: Uptake, biotransformation and elimination of selected pharmaceuticals in a freshwater invertebrate measured using liquid chromatography tandem mass spectrometry
Source: Chemosphere. 2017 Sep;183:389–400. doi: 10.1016/j.chemosphere.2017.05.083 (PMC5476196; doi:10.1016/j.chemosphere.2017.05.083)
Supplement: Supplementary file 1 [file mmc1.docx]

*Supplementary Information*

**UPTAKE, BIOTRANSFORMATION AND ELIMINATION OF SELECTED PHARMACEUTICALS IN A FRESHWATER INVERTEBRATE MEASURED USING LIQUID CHROMATOGRAPHY TANDEM MASS SPECTROMETRY**

*Thomas H. Miller^a^, Nicolas R. Bury^a^, Stewart F. Owen^b^, Leon P. Barron^a^**

*^a^Analytical & Environmental Sciences Division, Faculty of Life Sciences and Medicine, King’s College London, 150 Stamford Street, London, SE1 9NH, United Kingdom.*

*^b^AstraZeneca, Global Environment, Alderley Park, Macclesfield, Cheshire SK10 4TF, UK*

*Corresponding author email: [leon.barron@kcl.ac.uk](mailto:leon.barron@kcl.ac.uk);

Tel: +44 20 7848 3842; Fax: +44 20 7848 4980

Table of Contents

**S 1.0** Sample size reduction: matrix effects and recovery……………………………………….S3

**S 2.0** Minimised test design: estimation of bioconcentration factors……………………………S4

**S 3.0** Method linearity…………………………………………………………………………...S5

**S 4.0** Physico-chemical properties……………………………………………………………….S6

**S 5.0** Metabolite recovery and matrix effects……………………………………………………S7

**S 6.0** Steady-state BCF estimations……………………………………………………………...S7

**S7.0** LogD/P regressions with logBCF…………………………………………………………..S8

*List of Tables*

**Table S1.** Comparison of recoveries with a sample mass of 50 mg and 100 mg.

**Table S2.** Comparison of matrix effects with a sample mass of 50 mg and 100 mg.

**Table S3.** Estimation of bioconcentration factors using a full test design and a minimised test. SimBCF – simultaneous BCF, seqBCF – sequential BCF.

**Table S4.** Physico-chemical properties of the test compounds estimated relative to the pH of the exposure media.

**Table S5.** Recovery and matrix effects determined for the targeted metabolites.

**Table S6.** Mean [pharmaceuticals] at each time interval in the toxicokinetic exposures, (n=3).

*List of Figures*

**Figure S1.** Comparison of matrix matched calibration curves with(out) the addition of a stable isotope labelled-internal standard.

**Figure S2.** Comparison of log[BCF] and log*P* (top) and log*D* (bottom) of the pharmaceuticals selected in the toxicokinetic experiments.

**S 1.0** Sample size reduction: matrix effects and recovery

Table S1. Comparison of recoveries with a sample mass of 50 mg (n=6) and 100 mg (n=3).

|  | 100 mg | | 50 mg | |
| --- | --- | --- | --- | --- |
| Compound | Recovery (%) | RSD (%) | Recovery (%) | RSD (%) |
| Sulfamethazine | 41 | 5 | 37 | 20 |
| Trimethoprim | 65 | 2 | 91 | 13 |
| Metoprolol | 71 | 7 | 99 | 15 |
| Warfarin | 71 | 4 | 86 | 5 |
| Propanolol | 52 | 20 | 59 | 36 |
| Carbamazepine | 69 | 5 | 95 | 21 |
| Temazepam | 85 | 1 | 114 | 9 |
| Nimesulide | 87 | 5 | 64 | 8 |
| Nifedipine | 70 | 1 | 88 | 25 |
| Diazepam | 89 | 2 | 101 | 10 |

**Table S2.** Comparison of matrix effects with a sample mass of 50 mg (n=3) and 100 mg (n=3).

|  | 50 mg | | 100 mg | |
| --- | --- | --- | --- | --- |
|  | Matrix |  | Matrix |  |
| Compound | Effect (%) | SD | Effect (%) | SD |
| Sulfamethazine | -26 | 8 | -11 | 2 |
| Trimethoprim | -60 | 5 | -13 | 6 |
| Metoprolol | -32 | 14 | -11 | 2 |
| Propranolol | -74 | 13 | -56 | 4 |
| Carbamazepine | -63 | 16 | -9 | 2 |
| Warfarin | -23 | 19 | -28 | 4 |
| Nimesulide | -67 | 9 | -35 | 3 |
| Temazepam | -38 | 9 | -11 | 3 |
| Diazepam | -73 | 6 | -43 | 3 |
| Nifedipine | -53 | 18 | -39 | 4 |

**S 2.0** Minimised test design: estimation of bioconcentration factors

**Table S3.** Estimation of bioconcentration factors using a full test design and a minimised test. SimBCF – simultaneous BCF, seqBCF – sequential BCF.

|  | Full Design | | | Minimised Design | | |
| --- | --- | --- | --- | --- | --- | --- |
|  | SimBCF | SeqBCF^a^ | SeqBCF | SimBCF^b^ | SeqBCF^a^ | SeqBCF^b^ |
| Propranolol | 32 | 39 | 42 | 36 | 34 | 34 |
| Formoterol | 14 | 18 | 33 | 26 | 26 | 29 |
| Imipramine | 212 | 3811 | 4533 | 177 | 420 | 685 |
| Metoprolol | 16 | - | | 20 | - | |
| Terbutaline | 12 | 22 | 19 | 12 | 16 | 15 |
| Ranitidine | 17 | 81 | 112 | 30 | - | 9 |
| Diclofenac | 14 | 27 | 21 | 19 | 23 | 22 |
| Ibuprofen | 27 | 27 | 50 | 40 | 29 | - |

^a^sequential estimation using minitab

^b^Sequential estimation using linear regression

S 3.0 Method linearity


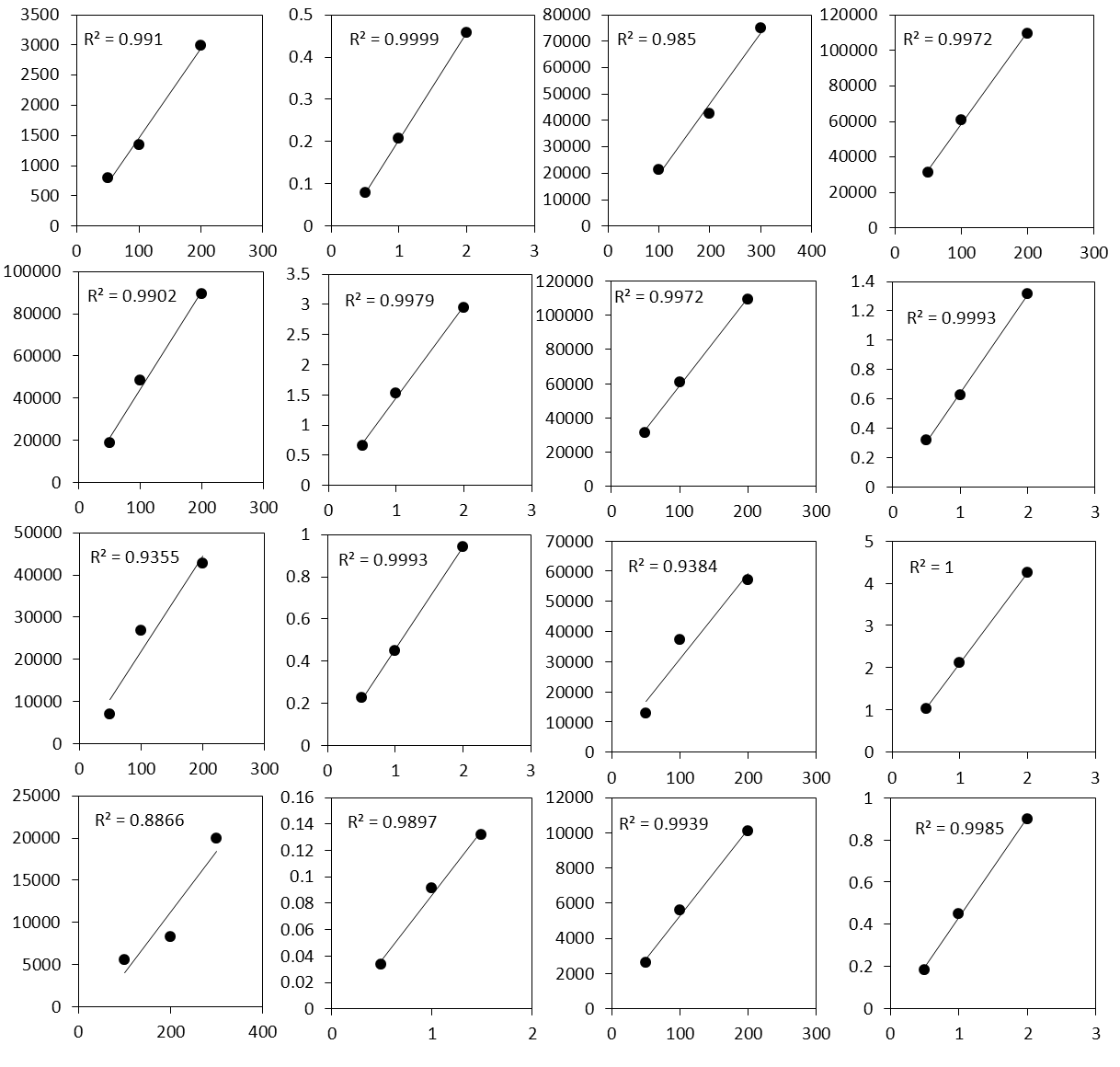


Warfarin

Warfarin - IS

Metoprolol - IS

Nifedipine - IS

Propranolol - IS

Trimethoprim - IS

Carbamazepine - IS

Sulfamethazine - IS

Diazepam - IS

Metoprolol

Nifedipine

Propranolol

Trimethoprim

Carbamazepine

Sulfamethazine

Diazepam


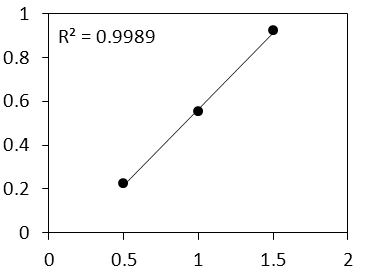


Trimethoprim-IS

**Figure S1.** Comparison of matrix matched calibration curves with/without the addition of a stable isotope labelled-internal standard.

**S 4.0** Physico-chemical properties

**Table S4.** Physico-chemical properties of the test compounds estimated relative to the pH of the exposure media.

| Compound | pH (n=4) | SD | Log*P* | Log*D* | Predominant Form |
| --- | --- | --- | --- | --- | --- |
| Carbamazepine | 8.09 | 0.03 | 2.28 | 2.3 | Neutral |
| Diazepam | 8.09 | 0.03 | 2.74 | 2.7 | Neutral |
| Temazepam | 8.5 | 0.12 | 2.12 | 2.1 | Neutral |
| Trimethoprim | 8.5 | 0.12 | 1.12 | 1.1 | Neutral |
| Nimesulide | 8.05 | 0.15 | 2.7 | 0.9 | Anionic |
| Nifedipine | 8.05 | 0.15 | 3.45 | 3.4 | Neutral |
| Sulfamethazine | 7.93 | 0.02 | 0.44 | 0.1 | Neutral/Anionic* |
| Warfarin | 8.2 | 0.08 | 3.11 | -0.2 | Anionic |
| Metoprolol | 8.2 | 0.08 | 1.85 | 0.5 | Cationic |
| Propranolol | 8.27 | 0.11 | 3.26 | 2 | Cationic |

logP/logD/ionic state – estimated from ACD Labs Percepta software

*1:1 ratio

**S 5.0** Metabolite recovery and matrix effects

**Table S5.** Recovery and matrix effects determined for the targeted metabolites (n=6) spiked 200 ng/g.

|  | Matrix |  | Recovery |  |  |
| --- | --- | --- | --- | --- | --- |
|  | Effect (%) | SD | (%) | SD | RSD (%) |
| CBZ-EPOX | 8 | 4 | 103 | 14 | 13 |
| HP-SULPH | -4 | 8 | 34 | 7 | 20 |
| HP | - | - | - | - | - |
| HP-GLU | - | - | - | - | - |
| OXAZ | 9 | 7 | 82 | 5 | 7 |
| NORDIAZ | -31 | 9 | 92 | 7 | 8 |

**S 6.0** Steady-state BCF estimations

**Table S6.** Mean [pharmaceuticals] at each time interval in the toxicokinetic exposures, (n=3).

|  | Concentration (ng g^-1^ dw) | | | | | | | |
| --- | --- | --- | --- | --- | --- | --- | --- | --- |
|  | 6h |  | 24h |  | 48h |  |  |  |
| Compound | Mean | SD | Mean | SD | Mean | SD | Steady State? | BCF_ss_ |
| Warfarin | 25 | 5 | 50 | 10 | 77 | 15 | No | - |
| Metoprolol | 6 | 1 | 11 | 2 | 9 | 2 | Yes | 9 |
| Nifedipine | 19 | 4 | 20 | 4 | 16 | 3 | Yes | 16 |
| Propranolol | 206 | 41 | 355 | 71 | 210 | 42 | * | 21 |
| Trimethoprim | 47 | 9 | 55 | 11 | 52 | 10 | Yes | 52 |
| Carbamazepine | 8 | 2 | 11 | 2 | 14 | 3 | Yes | 14 |
| Diazepam | 31 | 6 | 37 | 7 | 45 | 9 | Yes | 45 |
| Temazepam | 20 | 4 | 32 | 6 | 38 | 8 | * | 38 |


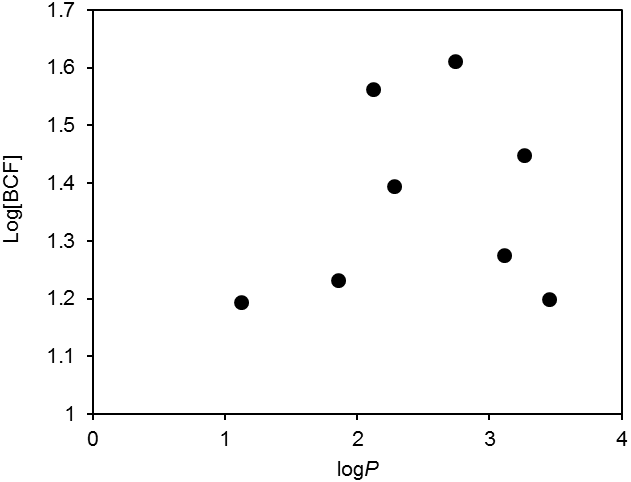

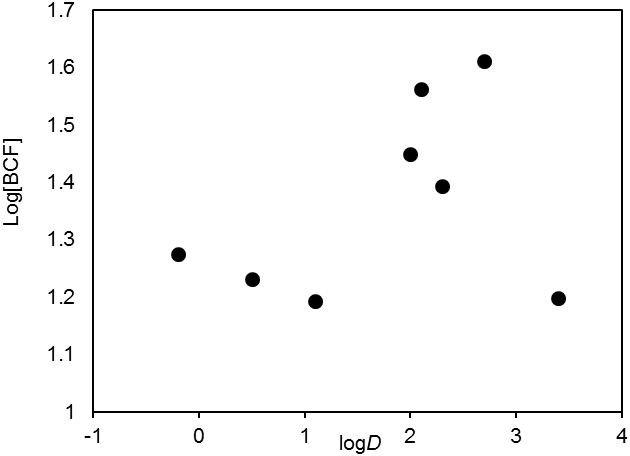


**Figure S2.** Comparison of log[BCF] and log*P* (top) and log*D* (bottom) of the pharmaceuticals selected in the toxicokinetic experiments.
